# Evaluating the Presence of a Stage IV Low-Grade Well-Differentiated Neuroendocrine Tumor of the Ileocecum: A Case Report with Evaluation of Staging Protocol of Neuroendocrine Tumors and Treatment Options Based on Current Available Evidence

**DOI:** 10.1155/2023/2919223

**Published:** 2023-08-17

**Authors:** Vineet Madishetty, Alicia J. Starr, Quyen D. Chu, P. A.-C. Brianna Starr

**Affiliations:** ^1^OMS-III-VCOM Carolina's, Spartanburg, SC, USA; ^2^Surgical Oncology, Orlando Health Institute, Orlando, FL, USA

## Abstract

Neuroendocrine tumors (NET) are rare neoplasms that can originate throughout the human body. An initial treatment option includes upfront surgical resection of the primary tumor (pT) if the tumor can be localized. Current systemic therapy options following resection of the pT or with evidence of metastatic disease include somatostatin analogs, evorlimus, peptide receptor radionuclide therapy, cytotoxic chemotherapy, and interferon alpha among other less common therapy options. We present a case of a patient with a NET that originated in the ileocecal region. The patient underwent upfront surgical resection with a right hemicolectomy due to the location of the tumor. The pT was notable for extensive invasion into the visceral peritoneum and metastasis to nearby lymph nodes. However, despite being diagnosed as a stage IV NET, the Ki67 index was less than 1%, categorizing it as a low-grade well-differentiated tumor. Following resection of the tumor, there was no evidence of metastasis to the liver on the follow-up magnetic resonance imaging and recurrent somatostatin receptor overexpressing neoplasm on the Gallium-68 DOTATE PET/CT scan. Due to the juxtaposition of the low grade of the tumor and the high staging, several different treatment options were discussed with the main distinction being whether to base these options off of the stage or the grade of the tumor in the case. Low-grade well-differentiated NET have a good prognosis. On the other hand, stage IV NET and tumors that have metastasized to nearby lymph nodes and organs have an increased likelihood to reoccur and worse outcomes. Recommendations for NET based on current evidence have a lack of clarity in terms of when to undergo observation versus systemic therapy.

## 1. Introduction

Neuroendocrine tumors (NET) are rare neoplasms that can be distributed widely throughout the body; however, there has been an increase in incidence particularly within the gastrointestinal system. The annual incidence of NETs is estimated to be 6.9/100,000 from 2012 using data from the Surveillance, Epidemiology, and End Results (SEER) program. The population-based study found a 6.4-fold increase in incidence from 1973 in the United States [1]. The SEER 17 database showed the most common gastroenteropancreatic NET primary sites in decreasing frequency are the rectum (17.7%), small intestine (17.3%), colon (10.1%), pancreas (7%), stomach (6%), and appendix (3.1%) [2].

NETs typically follow an indolent course and are therefore often diagnosed at later stages. Given the typically slow-growing nature of NETs, there can be a wide variety of symptoms, with vague abdominal pain being one of the most common presenting symptoms. If the tumor has grown significantly in size, a patient can also present with tumor-mass-related symptoms, such as obstruction of the gastrointestinal tract. Tumors arising in the right colon and ileocecal region are known to have a high capacitance and usually only produce symptoms once the tumor has reached >2 cm in size [3]. Additionally, in rare cases, tumors can be identified incidentally from routine colonoscopy, especially if located in the rectum and colon, or incidentally found on computerized tomography (CT) scans ordered for other clinical-related reasons [4]. Furthermore, due to the gastrointestinal systems rich lymphatic network in proximity, there is an increased probability for an NET tumor within the digestive system to metastasize to nearby lymph nodes and the liver [5].

Important features of NET prognosis include the Ki-67 proliferation index, nodal status, and anatomic location of the tumor [6]. The TNM staging system is used to help determine prognosis based on primary tumor (pT) size, lymph node metastasis, and any distant metastasis. However, the use of the Ki-67 and mitotic index is rising in prognostic value when determining the grade of the NET [7]. However, there is a lack of a standardized protocol for physicians to use when determining the plan of care and outlook for NET tumors based on TNM staging and histologic grade.

We present a patient who underwent a right hemicolectomy and mesenteric mass resection for a well-differentiated stage IV neuroendocrine tumor with a Ki67 index <1% in the ileocecal region.

## 2. Case Presentation

**Figure 1:**
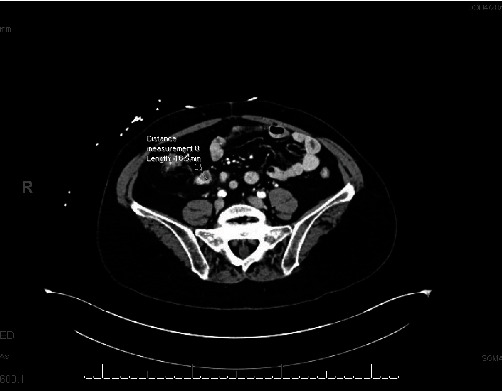
CT scan with intravenous contrast of the abdomen and pelvis showing an ileocecal mass.

A 75-year-old female was diagnosed with an ileocecal NET. Past surgical history was significant for an appendectomy over 20 years ago. Family history is significant for her mother having rectal cancer. The patient initially presented with complaints of progressively worsening right upper quadrant abdominal pain with radiation to her mid-back. She had associated nausea and lack of appetite. A CT scan of the abdomen/pelvis was performed and revealed a single enhancing mass in the ileocecal region that measured 2 cm × 3.2 cm. An adjacent enhancing lymph node measuring 2.2 cm × 1.7 cm × 1.4 cm and a spiculated mesenteric lesion in the right lower abdomen measuring 2.2 cm × 1.7 cm × 1.4 cm were also found. CT of the abdomen and pelvis with IV contrast is detailed in Figure 1.

The patient was referred to a medical oncologist for suspicion of a carcinoid with local spread. A body PET/CT scan was obtained beforehand and noted a hypermetabolic lesion in the cecal region along with several small ileocolic lymph nodes.

A colonoscopy was performed, which revealed a firm, friable mass in the terminal ileum, which was biopsied. The mass was reported to cause deformity in the terminal ileum, cecum, and proximal ascending colon. The pathology report showed a well-differentiated NET, World Health Organization (WHO) Grade I. Lab values were significant for elevated serotonin at 805 ng/ml and chromogranin A at 211.9 ng/ml.

The patient was presented to the Multidisciplinary GI Tumor Board, which included representatives from medical oncology, research, palliative care, radiation oncology, surgical oncology, navigation, radiology, and pathology. The imaging and pathology were reviewed by the board, and the consensus was that the imaging showed an arterially enhancing lesion at the ileocecal valve with small adjacent lymph nodes, all of which show uptake on PET/CT. There was also a mesenteric mass that was not PET-avid. Pathology showed well-differentiated neuroendocrine tumor with Ki67 of 1%. The recommendation was to proceed with a right hemicolectomy.

The surgical oncologist performed a right hemicolectomy along with mesenteric mass resection. The surgical pathology report revealed a final diagnosis of:

- Grade 1, well-differentiated neuroendocrine cell neoplasm, 2.6 cm in size of the terminal ileum with invasion onto the serosal surface and extension to the radial margin. Other findings included:
- Extensive mesenteric adipose tissue tumor deposits.
- Four out of fifteen mesenteric lymph nodes were positive for metastatic tumor.

The tumor extensively infiltrated the muscularis propria with invasion of the mucosa and peri-ileal adipose tissue. The tumor further extended onto the serosal surface (visceral peritoneum). The tumor cells were well-differentiated and contained rare mitosis. The immunohistochemistry of the tumor cells showed positive staining for pancytokeratin, CgA, CD56, synaptophysin, villin, and CDX-2. The Ki-67 index was less than 1%.

The pT category of the tumor was identified as pT4. The regional lymph node (pN) category of the tumor was identified at pN1. The histologic type and grade were identified as a G1, well-differentiated neuroendocrine tumor. Postoperatively, the patient tolerated the procedure very well and was discharged on postoperative day 7 once bowel function had returned, and the patient was tolerating a regular diet.

After reviewing the final diagnostic pathology report, the patient was presented again to the Multidisciplinary GI Tumor Board. There was extensive discussion that if there is nodal involvement or Ki67, it should dictate that the patient should be observed versus undergoing systemic therapy. The final recommendations were to obtain a magnetic resonance imaging (MRI) liver protocol to assess for any metastatic disease and PET/CT Ga-67 DOTATE scan for baseline.

**Figure 2:**
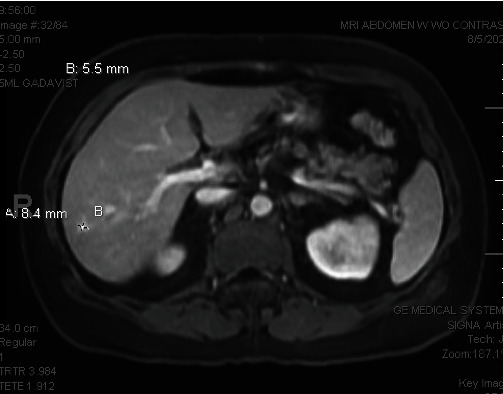
MRI of the abdomen without contrast. Image provided courtesy of Orlando Health.

**Figure 3:**
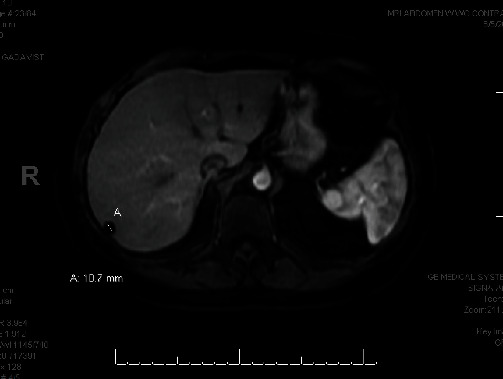
MRI of the abdomen with contrast showing benign small cysts in the right lobe of the liver.

On the MRI of the abdomen with/without contrast, benign small cysts were noted in the right lobe of the liver in segments 7 and 6 detailed in Figures 2 and 3. There was a small, stable, focal enhancing lesion in the right lobe liver suggestive of flash-filling hemangioma.

On the nuclear medicine PET/CT skull base to mid-thigh Gallium-68 DOTATE PET CT scan, there was no evidence of recurrent somatostatin receptor (SSTR) overexpressing neoplasm.

In conclusion, the patient has well-differentiated pT4N1 neuroendocrine tumor, Ki67 < 1% with extensive mesenteric tumor deposits with positive radial margin and no evidence of metastatic disease to the liver or evidence of recurrent SSTR overexpressing neoplasm. However, the question remains, should the patient undergo expectant observation, or should the patient start systemic therapy?

## 3. Discussion

Considering the rarity of NETs, and how it is often diagnosed at later stages due to their aforementioned indolent course and vague presenting symptoms, there is a lack of information and consensus from medical professionals on how to appropriately treat and stage these type of tumors. There has been much controversy surrounding optimal treatment for patients with NETs due to the lack of randomized control trials to the ability to accurately define it. This leads to many multifaceted discussions of how to properly treat these types of tumors and further complicates the goal of standard treatment [8].

The case is also notable because the NET is located in the ileocecal region. NET originating from the ileum is one of the most common locations. However, NETs originating from the cecum only consist of about 3.07% of all NETs in the United States [2]. Approximately 7.5–10% of NETs originate from the colon as a whole [2, 9]. NETs are already rare neoplasms, but there is a further lack of knowledge on proper prognosis and treatment protocol of NETs originating from the colon due to the low percentage of incidence compared with other pT origins. Additionally, it is unclear what staging protocol to use when the tumor consists of the ileocecal region given that AJCC TNM staging guidelines are differentiated between the small intestine and colon as being two separate guideline protocols. The most updated 8th edition AJCC TNM staging system for NET of the jejunum and ileum is shown in Table 1 and of the colon in Table 2. The carcinoid tumor in this case report is a pT4N1 neuroendocrine tumor. However, using the AJCC guidelines for prognostic staging, the tumor would be considered stage III using the colon TNM staging protocol, versus stage IV using the jejunum and ileum staging protocol. Precise prognostic staging of the tumor in the case study cannot be met due to a lack of criteria for NET involving more than one region.

Furthermore, the grade of the tumor is also clinically significant in regard to the prognosis of the tumor. The WHO determines the grade of NETs of the gastrointestinal tract based on Ki-67 and mitotic index. Applying the 5th edition WHO grading criteria for NETs in Table 3, the tumor in the case study is a low-grade well-differentiated NET. When considering the rate of overall survival (OS) for NET, the low grade of the tumor would be considered to have a good prognosis. However, the TNM staging of the tumor and the extent of the invasion into nearby organs would be considered a poor prognosis for this case.

The outlook of a patient's prognosis for NETs has been shown to vary from prognostic staging, the extent of invasion of the tumor, histological grade, and pT location. The median OS for NETs, regardless of stage and grade, was reported to be 9.3 years. Grade 1 NET has a median OS of 16.2 years, grade 2 of 8.3 years, and grade 3 being 10 months. Additionally, the median OS was >30 years for localized NET, regional NET being 10.2 years, and distant NET being 12 months (*P* < 0.001) [1]. Prognosis depending on the pT site also differs with some tumor origins having a better prognosis. Tumors originating from the colon have a worse prognosis compared with the small intestine, and other nearby locations including the appendix and rectum [9]. In a study using the SEER Registry from 1973–2014, survival rates of patients with carcinoid tumors were examined based on the pT site. The small intestine showed 81.3%, 60.6%, and 44.2% survival rates for 1, 3, and 5 years incongruent order. In contrast, the colon showed 72.3%, 53.3%, and 40.9% survival rates for 1, 3, and 5 years [10]. In another SEER-based analysis from 1992 to 1999, the colon was further specified by location and showed similar 5-year survival rates for the cecum and colon, excluding the rectum (61% and 61.8% for 5-year survival for all stages) [11]. The patient presented in this case report had a significant surgical history of an appendectomy. This can exclude the pT originating from the appendix, which also has differing prognoses based on location. However, in this case, the ileocecal region overlaps prognosis determination from the colon and small intestine.

Questions are also raised about the frequency of post-treatment follow-up to look for recurrence of the NET due to limited evidence. However, The National Comprehensive Cancer Network recommends abdominal CT or MRI for gastrointestinal NETs every 3–12 months postresection. After 1-year postresection, a follow-up CT scan or MRI is recommended every 1–2 years. Assays of biochemical markers for each follow-up visit are also indicated. After 10 years, continued surveillance is based on clinical indications [12, 13]. However, the slow-growing nature of NETs can indicate a focus on long-term surveillance, in contrast to frequent follow-ups for recurrence [14]. For example, a previous study evaluated the disease-free interval of patients with a small bowel NET following resection. The median disease-free interval was 5.5 years. Those with lymph node involvement at the time of resection had a greater incidence of recurrence compared with those without lymph node involvement. 37 of 98 study participants had a recurrence of the NET with lymph node involvement [15]. Considering that recurrence can occur well after 5 years from the initial diagnosis, it is important to continually monitor for clinical symptoms related to these types of tumors.

Treatment options for NETs after resection of the tumor are not clear and many treatment options are still being studied for their effectiveness. However, with the increased incidence of NETs, more research on treatment options and standardized approaches to these tumors should be further investigated. Somatostatin analogs (SSA) are currently considered one of the first-line treatment options for NETs. SSA is often used for anti-proliferative effects as well as symptomatic control [25]. The PROMID study group performed a phase IIIb study in patients with well-differentiated metastatic mid-gut NETs or inoperable NETs. A patient was either given a placebo or octreotide LAR 30 mg (SSA) intramuscularly in monthly intervals. The study found that octreotide LAR has lengthens the time of tumor progression compared with the placebo group. Both non-functioning and functioning NETs responded similarly. In addition, an antiproliferative response was more evident in patients with a low-hepatic load and with a resected pT [16].

The CLARINET trial examined the use of lanreotide (another SSA) in patients with well-differentiated NET of grade 1 or grade 2. The study concluded that lanreotide was associated with a prolonged progression-free survival interval for patients with metastatic NET of grade 1 or grade 2 [17]. However, it is important to consider whether or not the patient will be on long-term SSA therapy before resection of the pT. The question arises of whether or not to perform a cholecystectomy during the primary resection of NET. Chronic treatment with SSA is known to cause gallstones and decrease overall gallbladder function [26]. This consideration has many factors involved, including the age of the patient, the probability they will need a future cholecystectomy, and the likelihood they will require SSA therapy and the duration of it. Future research should be considered on this topic due to many factors influencing making an appropriate decision on this surgical approach [8]. There is a lack of definitive protocol, that is, widely accepted on this topic, and could be further investigated.

Other second-line treatment options have been evaluated, such as everolimus, peptide receptor radionuclide therapy (PRRT), interferon-alpha, and cytotoxic chemotherapy [18, 19]. Evorlimus was considered more of a third- to fourth-line treatment option for patients with extensive and aggressive tumor infiltration or spread. This would indicate that tumors with a higher mitotic index, which goes along with being termed aggressive, and rapidly dividing could be considered for this treatment option.

PRRT therapy uses radiolabeled SSA that target tumors expressing SSTR to deliver targeted radiation to tumors. The mechanism of action of PRRT therapy involves the linking of radiolabeled SSA (made up of an isotope radionuclide, a carrier molecule, often derived from octreotide, and a chelating agent, which aims to stabilize the complex), which are then internalized, degraded, and stored in lysosomes [23]. The radioactive compounds are then released inside the cells. This selectivity for SSTR seems to be why PRRT therapy may have lower toxicity in comparison with other anti-cancer therapeutic options [23]. This treatment option is highly dependent upon whether SSTR are expressed. NETTER-1 study conducted a phase 3, randomized trial was done using 177Lu-Docusate, which is part of PRRT therapy. The results of this trial showed longer progression-free survival compared with high-dose octreotide for mid-gut NETs. Of those in the clinical trial, in 73% of patients, the pT was located in the ileum. 83% of patients had metastasis to the liver and 62% to the lymph nodes or both for some patients. Each grade of the tumor was evenly balanced in the placebo versus treatment group. In addition, approximately 80% of patients had previous surgical resection of the tumor in each group. The study reported limited acute toxic effects in the patients who were given the treatment [22].

A cohort study of 508 patients with enteropancreatic neuroendocrine tumors compared with peptide receptor radionuclide therapy to the standard therapy of SSA, and disease progression was monitored. The study data showed that patients who had disease progression with SSA therapy had experienced significantly approved survival outcomes when compared with patients who received upfront chemotherapy or targeted therapy. Studies have indicated that PRRT therapy has proven effective in treating gastroenteropancreatic neuroendocrine tumors with high SSTR expression [25].

There is great potential in considering PRRT as an early treatment option as it is quite efficacious in terms of treating advanced stages of intestinal NET's, which are unamenable to therapy with SSA. This method also has limited toxicity in comparison with other modalities, which can make treatment with PRRT a very beneficial option. This therapy is highly effective in patients who overexpress SSTR. However, further randomized control trials and studies still need to be conducted to further investigate and standardize a treatment protocol for the timing of when to use PRRT [23–25].

Interferon-alpha has also been explored and is thought to be used as an add-on therapy if patients are resistant to octreotide therapy [19]. However, interferon-alpha treatment has had conflicting evidence in studies. Some studies showed increased toxicity without therapeutic benefits, whereas others showed benefits when combined with SSA, but small patient numbers should be considered a factor when interpreting the results [20].

Chemotherapy is often reserved for high-grade NET and is not effective in low-grade well-differentiated NETs [8]. Tumor grade (Ki-67 and mitotic index) influences the efficacy in response to chemotherapy, most likely due to chemotherapy historically known to target rapidly dividing cells. An analysis reported a 60% response rate to high-grade tumors, compared with 14% for low grade and 33% for an intermediate grade. However, due to the high toxicity associated with chemotherapy, this treatment option is highly controversial as to whether or not this treatment option is to be considered to be beneficial to the patient. Although, the grade of the NET help predict response to chemotherapy [21].

## 4. Conclusion

There is a shortage of information on whether or not to use the grading system or TNM staging guidelines when looking at potential treatment options. This case brings interest considering the extensive invasion of the tumor into the nearby serosa, along with a very low Ki-67 index. Evidence of survival and reoccurrence rate differs between grades as well as differences in the extent of invasion of the tumor. This arising the question of how exactly would you determine, which treatments to base it on to prevent the tumor to reoccur and to extend the survival length. There is evidence to show that grade 1 tumors have a good prognosis, whereas stage III/IV NETs have worse. NETs are known to be rare and can originate in a variety of locations throughout the body, which can further add to an unpredictable expectation of these types of tumors. Although researchers have been gathering information to try to classify and treat these types of rare tumors, there are still vague guidelines on when it would be beneficial to treat the patient based on prognosis and the primary location of the tumor. The increasing incidence of NET's should also encourage future studies to solidify clinical management recommendations based on the classification of the staging and grade of the diagnosis.

## Figures and Tables

**Table 1:** NET of the jejunum and ileum TNM staging AJCC UICC 8th edition.

Primary tumor (T)		Regional lymph nodes (N)		Distant metastasis (M)	
T category	T criteria	N category	N criteria	M category	M criteria
T0	No evidence of a primary tumor	N0	No regional LN metastasis	M0	No distant metastasis
T1	Tumor invades lamina propria or submucosa and <1 cm or equal to	N1	Regional LN metastasis of less than 12 nodes	M1	Distant metastasis
T2	Tumor invades muscularis propria or >1 cm	N2	Large mesenteric masses >2 cm and/or >12 nodal deposits		
T3	Tumor invades into subserosal tissue without penetration of overlying serosa				
T4	Tumor invades the visceral peritoneum or other organs and adjacent structures				

**Table 2:** NET of the colon and rectum TNM staging AJCC UICC 8th edition.

Primary tumor (T)		Regional lymph nodes (N)		Distant metastasis (M)	
T category	T criteria	N category	N criteria	M category	M criteria
T0	No evidence of a primary tumor	N0	No regional lymph node metastasis	M0	No distant metastasis
T1	Invades lamina propria or submucosa and is less than or equal to 2 cm	N1	Regional lymph node metastasis	M1	Distant metastasis
T2	Invades muscularis propria or is >2 cm with invasion of the lamina propria or submucosa				
T3	Invades through muscularis propria into subserosal tissue				
T4	Tumor invades visceral peritoneum or other adjacent structures				

**Table 3:** Classification and grading of well-differentiated NET of the gastrointestinal tract (World Health Organization, 2019) [27].

Terminology	Grade	Mitotic index (mitoses/2 mm^2^)	Ki-67 index (%)
NET, G1	Low	<2	<3
NET, G2	Intermediate	2–20	3–20
NET, G3	High	>20	>20

## Data Availability

The data used to support the findings of this study are included within the article. No underlying data was collected or produced in this study.
